# Peat moisture dataset of Sumatra peatlands

**DOI:** 10.1016/j.dib.2023.108889

**Published:** 2023-01-11

**Authors:** Muh Taufik, Marliana Tri Widyastuti, I Putu Santikayasa, Chusnul Arif, Budiman Minasny

**Affiliations:** a Department of Geophysics and Meteorology, IPB University, Jalan Meranti Wing 19 Lvl 4 Darmaga Campus, Bogor 16680, Indonesia; b School of Life and Environmental Science, Sydney Institute of Agriculture, The University of Sydney, Sydney, New South Wales 2006, Australia; c Department of Civil and Environmental Engineering, IPB University, Darmaga Campus, Bogor 16680, Indonesia

**Keywords:** Soil moisture content, Field monitoring, Human modified, Tropical peatland, Water retention

## Abstract

Peatland is a unique ecosystem that is key in regulating global carbon cycle, climate, hydrology, and biodiversity. Peat moisture content is a key variable in ecohydrological and biogeochemical cycles known to control peatland's greenhouse gas emissions and fire vulnerability. Peat moisture is also an indicator of the success of peat restoration projects. Here we present datasets of peat moisture dynamic and retention capacity of degraded tropical peatlands. The data were collected from automatic daily monitoring and field campaigns. The peat moisture content data consists of daily data from 21 stations across three peatland provinces in Sumatra Island, Indonesia, from 2018 to 2019. In addition, peat water retention data were collected from field campaigns in Riau province. This dataset represents human modified peatlands which can be used as a benchmark for hydrological and biogeochemical models.

**Specifications Table**

**Table:** 

Subject	Environmental Sciences, Earth and Planetary Sciences
Specific subject area:	Hydrology, Soil Science.
Type of data:	Figure, Table
How the data were acquired:	Station measurement, field sampling
Data format:	Raw, Analysed
Description of data collection:	Peat moisture content from 21 field stations in three peatland provinces in Indonesia, collected using automatic measurement. Water retention data of 105 peat samples from 15 plots in Riau provinces. The samples represent topsoil layer (0-30 cm) and sub-soil layer (50-70 cm)
Data source location:	Riau, Jambi, and South Sumatra peatlands, Indonesia. Geographical extent ranges from 100.12^0^ to 106.10^0^ E longitude and 2.71^0^ N to 4.16^0^ S latitude
Data accessibility:	Data available within the article and in the Mendeley Data: Moisture data: https://data.mendeley.com/datasets/27y943wbkw, DOI: 10.17632/27y943wbkw.1 Water retention data: https://data.mendeley.com/datasets/jj35xcx98v, DOI: 10.17632/jj35xcx98v.1 https://data.mendeley.com/datasets/9stvfxystv, DOI:10.17632/9stvfxystv.1

## Value of the Data

• Moisture content of tropical peatlands determines CO_2_ flux and their vulnerability to fire. However, public datasets at daily levels are rare.
• Hydrologists and environmentalists can use the datasets as a benchmark in modelling tropical peatlands. In addition, the datasets provide a unique insight into peat moisture dynamics of degraded peatland as affected by management.
• The knowledge generated from this dataset can help to improve restoration project activities in tropical peatlands.
• The dataset could also be used for drought-fire modelling in tropical peatlands [1], and for supporting the development of database on Indonesian's peat physical properties [2], especially water retention [3], and validating global wetland models.

## Objective

This paper provides the first of its kind moisture data on tropical peatlands, which can be used as a benchmark for modelling and further research in the field.

## 1 Data Description

Peatlands are unique ecosystems that store up to one-third of the world's soil organic carbon stock. Conversion of tropical peatlands to agriculture via drainage has led to the rapid depletion of C stocks and made them vulnerable to fire. Consequently keeping peatlands wet is crucial to restoring their condition. Moisture content of peats is a key variable that determines peatlands’ ecohydrological and biogeochemical functions. Peat moisture is known to control greenhouse gas emissions and vulnerability to fire [1,4]. In addition, peat moisture is also a key indicator in determining the success of peat restoration activities, which are expandingly being applied in Indonesian peatlands. However, dynamic moisture data on tropical peatlands moisture rare.

**Fig. 1:**
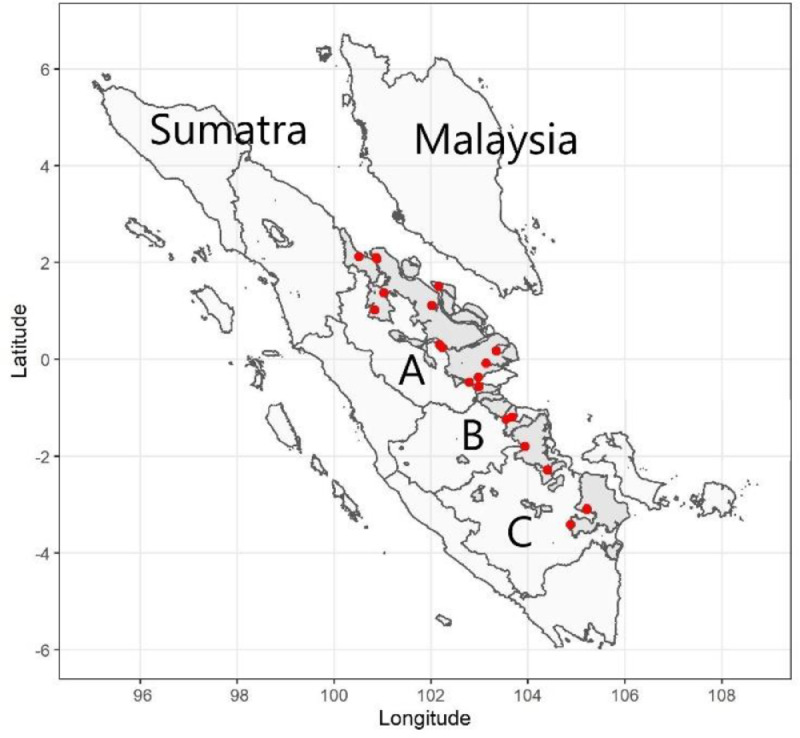
Location of field stations for peat moisture monitoring in the three provinces of Indonesian peatland: (A) Riau, (B) Jambi, and (C) South Sumatra. Grey color indicates peat, while red dots show the monitoring locations.

This paper reports unique datasets of peat moisture dynamics and moisture characteristics data from lowland peatlands in Sumatra, Indonesia. More specifically, we provide daily moisture content of the upper peat layer. Fig. 1 presents the peatland area on the east coast of Sumatra, where the monitoring sites were located. Data from 21 field stations were presented, where 16 sites ae in Riau, 2 in Jambi, and 5 in South Sumatra. The summary statistics of peat moisture content for each station are presented in Table 1. The summary shows mean peat water content also under dry and wet conditions for 21 peatlands in 2018-2019. The moisture ranges from 11.3% to 80% (volume percentage).

**Table 1:** Summary statistics of peat moisture dynamics in Sumatra peatland, Indonesia. Code refers to station name. The summary was calculated for the period of 2018-2019. Peat hydrological unit represents peat ecosystem between two river, or between river and sea, or within a swamp.

No	Code	Peat Hydrological Unit	Long	Lat	Province	Moisture content (% volume)		
						Mean	Min	Max
1	BRG_140302_01	KHG Pulau Bengkalis	102.16	1.51	Riau	46.2	19.7	73.6
2	BRG_140312_02	KHG Sungai Rokan-Sungai Siak Kecil	102.02	1.11	Riau	69.8	44.3	78.1
3	BRG_140402_01	KHG Sungai Enok-Sungai Batang	102.99	-0.57	Riau	43.8	31.1	57.4
4	BRG_140405_02	KHG Sungai Gaung-Sungai Batangtuaka	102.79	-0.48	Riau	29.4	12.2	73.3
5	BRG_140411_02	KHG Sungai Gaung-Sungai Batangtuaka	102.98	-0.37	Riau	36.7	26.0	56.9
6	BRG_140412_01	KHG Sungai Kampar-Sungai Gaung	103.13	-0.08	Riau	49.1	36.3	59.7
7	BRG_140415_01	KHG Sungai Kampar-Sungai Gaung	103.35	0.18	Riau	39.1	24.4	55.4
8	BRG_140508_02	KHG Sungai Kiyap-Sungai Kerumutan	102.23	0.24	Riau	45.9	24.3	74.6
9	BRG_140508_03	KHG Sungai Kiyap-Sungai Kerumutan	102.18	0.31	Riau	31.9	12.1	70.8
10	BRG_140613_02	KHG Sungai Rokan Kiri-Sungai Mandau	100.84	1.02	Riau	52.7	22.4	79.9
11	BRG_140701_02	KHG Sungai Barumun-Sungai Kubu	100.51	2.12	Riau	61.2	33.3	79.9
12	BRG_140702_02	KHG Sungai Rokan-Sungai Siak Kecil	100.89	2.08	Riau	33.6	11.9	79.7
13	BRG_140702_03	KHG Sungai Rokan-Sungai Siak Kecil	100.87	2.11	Riau	43.9	16.8	79.9
14	BRG_140713_01	KHG Sungai Rokan-Sungai Siak Kecil	101.03	1.38	Riau	30.0	13.7	73.8
15	BRG_150709_02	KHG Sungai Mendahara-Sungai Batanghari	103.54	-1.24	Jambi	42.5	34.8	55.9
16	BRG_150710_02	KHG Sungai Mendahara-Sungai Batanghari	103.67	-1.19	Jambi	59.5	31.3	78.0
17	BRG_160205_01	KHG Sungai Burnai-Sungai Sibumbung	104.88	-3.42	South Sumatra	40.0	11.8	79.7
18	BRG_160214_01	KHG Sungai Saleh-Sungai Sugihan	105.22	-3.11	South Sumatra	32.8	14.9	69.4
19	BRG_160214_02	KHG Sungai Saleh-Sungai Sugihan	105.22	-3.09	South Sumatra	60.3	37.3	78.1
20	BRG_160609_01	KHG Sungai Lalan-Sungai Merang	103.93	-1.80	South Sumatra	36.7	11.3	80.0
21	BRG_160611_01	KHG Sungai Sembilang-Sungai Lalan	104.41	-2.28	South Sumatra	40.1	18.7	72.4

In addition to peat moisture, we collected peat soil samples in Riau to obtain their water retention characteristics (see Methods section). Table 2 presents peat water retention data for two peat layers from 19 plots with various human-modified land-uses. Water retention of peats controls hydrological processes and determines the resilience of peatlands to fire risk [3,5]. For example, in the calculation of drought fire index developed in tropical peatland, water retention controls the contribution of the groundwater table to the moisture content of soil surface [1,3]. Unfortunately, water retention data on peats are rare. With the realisation of the importance of peat hydrology, there is a global need for a hydraulic properties dataset to allow the development of pedotransfer functions for global hydrology modelling.

**Table 2:** Peat moisture content (%) at various water suction and land-uses in Riau province. Layer refers to the depth of peat sample; 0-30cm for topsoil and 50-70cm for subsoil. Land-use shows dominant landscape around the station. n indicates the number of samples. pF represents for water retention suction levels.

No	Plot	Layer	land-use	n	Moisture content (% vol)				Sampling date
					pF1 (-1 kPa)	pF2 (-10 kPa)	pF2.54 (-33 kPa)	pF4.2 (-1500 kPa)	
1	plot 1	topsoil	Rubber	3	63.8	42.9	35.6	11.9	2019-08-28
2	plot 1	subsoil	Rubber	3	58.0	36.4	30.7	12.2	2019-08-28
3	plot 2	topsoil	Mixed garden	3	65.4	43.5	37.2	15.0	2019-08-28
4	plot 2	subsoil	Mixed garden	3	65.8	45.7	39.5	14.8	2019-08-28
5	plot 3	topsoil	Oil Palm	3	67.4	44.4	37.4	15.7	2019-08-28
6	plot 3	subsoil	Oil Palm	3	64.1	42.1	35.7	14.1	2019-08-28
7	plot 4	topsoil	Rubber	2	72.7	47.3	39.7	15.4	2019-08-29
8	plot 4	subsoil	Rubber	2	65.1	42.0	35.9	13.6	2019-08-29
9	plot 5	topsoil	Mixed garden	2	75.0	51.8	43.6	16.9	2019-08-29
10	plot 5	subsoil	Mixed garden	2	66.9	43.8	35.5	13.4	2019-08-29
11	plot 6	topsoil	Oil Palm	2	69.6	47.3	40.7	16.7	2019-08-29
12	plot 6	subsoil	Oil Palm	2	64.9	43.9	36.6	11.8	2019-08-29
13	plot 7	topsoil	Shrub	3	63.2	42.5	34.1	15.1	2019-11-01
14	plot 8	topsoil	Oil Palm	3	61.6	41.0	33.8	17.1	2019-11-01
15	plot 8	subsoil	Oil Palm	3	55.9	40.1	31.9	16.3	2019-11-01
16	plot 9	topsoil	Cropland	3	64.9	42.5	34.2	15.2	2019-11-01
17	plot 9	subsoil	Cropland	3	63.1	48.3	40.7	15.4	2019-11-01
18	plot 10	topsoil	Cropland	3	62.5	49.7	41.6	15.7	2019-11-02
19	plot 10	subsoil	Cropland	3	63.4	47.8	40.3	14.1	2019-11-02
20	plot 11	topsoil	Shrub	4	66.4	46.0	38.8	16.0	2019-11-03
21	plot 11	subsoil	Shrub	4	58.7	42.7	35.0	12.3	2019-11-03
22	plot 12	topsoil	Oil Palm	4	76.1	53.4	46.5	22.1	2022-09-28
23	plot 12	subsoil	Oil Palm	4	74.4	54.3	48.0	23.3	2022-09-28
24	plot 13	topsoil	Oil Palm	1	73.8	50.2	43.6	21.8	2022-09-28
25	plot 13	subsoil	Oil Palm	1	77.0	52.7	45.0	19.9	2022-09-28
26	plot 14	topsoil	Oil Palm	4	73.4	50.4	43.9	21.5	2022-09-28
27	plot 14	subsoil	Oil Palm	4	78.0	53.4	46.8	21.1	2022-09-28
28	plot 15	topsoil	Oil Palm	3	77.5	49.1	43.1	19.7	2022-09-29
29	plot 15	subsoil	Oil Palm	3	74.5	50.3	44.9	18.9	2022-09-29
30	plot 16	topsoil	Oil Palm	3	77.4	53.9	47.0	23.3	2022-09-29
31	plot 16	subsoil	Oil Palm	3	76.8	52.8	46.3	22.3	2022-09-29
32	plot 17	topsoil	Imperata	1	70.1	43.0	35.0	11.5	2022-09-30
33	plot 17	subsoil	Imperata	1	78.4	48.2	39.2	13.2	2022-09-30
34	plot 17	topsoil	Oil Palm	1	77.5	58.5	53.0	23.1	2022-09-30
35	plot 17	subsoil	Oil Palm	1	79.1	59.7	55.6	24.8	2022-09-30
36	plot 17	topsoil	Rubber	2	80.5	52.8	45.2	21.5	2022-09-29
37	plot 17	subsoil	Rubber	2	75.9	48.8	41.2	18.1	2022-09-29
38	plot 18	topsoil	Imperata	1	83.6	58.6	50.1	18.2	2022-09-30
39	plot 18	subsoil	Imperata	1	81.4	56.9	49.4	20.5	2022-09-30
40	plot 18	topsoil	Oil Palm	1	82.4	62.7	56.8	25.5	2022-09-30
41	plot 18	subsoil	Oil Palm	1	79.9	60.9	55.9	27.0	2022-09-30
42	plot 19	topsoil	Imperata	1	81.3	58.0	50.0	23.4	2022-09-30
43	plot 19	subsoil	Imperata	1	78.8	55.7	48.7	20.8	2022-09-30
44	plot 19	topsoil	Pinapple	2	81.6	56.4	48.1	19.7	2022-09-30
45	plot 19	subsoil	Pinapple	2	83.6	58.7	49.6	24.9	2022-09-30

## 2 Experimental Design, Materials and Methods

### 2.1 Study Sites

We collected data from human-modified peatlands in Indonesia. The location for sampling is presented in Fig. 1. According to the Köppen classification, the climate is a tropical rain forest [7] with average annual rainfall between 2500 and 3000 mm [1,8].

Peatlands in Sumatra are hotspots of land-use change [6] as the drastic transformation has converted pristine swamp forests into economic-oriented plantations. The peatlands are mostly utilised for agriculture (>60%) such as small-holder and industrial plantations [6], while the proportion of pristine swamp forests is less than 10%. Fires in the study site were frequently reported in response to peatland degradation and intensive drainage [9].

### 2.2 Peat Moisture Monitoring

At each of the 21 stations, peat moisture content was measured using HydraProbe sensor (Stevens Water, America) at a depth of 10-20 cm. The unit of measurement is in percentage volume of water. This depth was selected as it represents the level of peat dryness that determines the easiness of fire ignition [10]. The sensor recorded moisture on an hourly basis, and here was reported as a daily average moisture value from 15 Oct 2018 to 20 December 2019. We processed the peat moisture data to obtain the summary statistics for each station using R statistical software [11].

### 2.3 Peat Samples for Water Retention Analysis

To understand the peat's physical properties, we conducted three field campaigns to collect peat samples from various land uses in Riau. The first campaign was at August 2019, then followed by the 2^nd^ campaign in November 2019 with 59 samples were collected in 2019. In the end of September 2022, we carried out the third field campaign to collect 48 peat samples. The samples were taken from two depths using 105 cm^3^ metal cylinders (height: 5 cm, diameter: 5.17 cm), representing the topsoil-layer (0–30 cm) and subsoil-layer (50–70 cm). The samples were analysed for water retention at near saturation or pF1 (-1 kPa), pF2 (-10 kPa), pF2.54 or field capacity (-33 kPa), and pF4.2 or wilting point (-1500 kPa) using the pressure plate method [12].

## Ethics Statements

Our work does not involve studies with animals and humans.

## CRediT authorship contribution statement

**Muh Taufik:** Conceptualization, Methodology, Investigation, Data curation, Writing – original draft, Visualization. **Marliana Tri Widyastuti:** Conceptualization, Methodology, Investigation, Data curation, Writing – original draft. **I Putu Santikayasa:** Conceptualization, Methodology, Investigation. **Chusnul Arif:** Conceptualization, Methodology, Investigation. **Budiman Minasny:** Writing – review & editing.

## Declaration of Competing Interest

The authors declare that they have no known competing financial interests or personal relationships that could have appeared to influence the work reported in this paper.

## Data Availability

Station-based peat moisture in Sumatra peatland (Reference data) (Mendeley Data).
